# Result Publication of Chinese Trials in World Health Organization Primary Registries

**DOI:** 10.1371/journal.pone.0012676

**Published:** 2010-09-13

**Authors:** Liu Xuemei, Li Youping, Yin Senlin, Song Shangqi

**Affiliations:** 1 Chinese Journal of Evidence-Based Medicine, West China Periodicals Press, West China Hospital of Sichuan University, Chengdu, China; 2 Chinese Evidence-Based Medicine Center, West China Hospital of Sichuan University, Chengdu, China; 3 West China School of Medicine of Sichuan University, Chengdu, China; University Paris Descartes, France

## Abstract

**Background:**

Result publication is the key step to improve the transparency of clinical trials.

**Objective:**

To investigate the result publication rate of Chinese trials registered in World Health Organization (WHO) primary registries.

**Method:**

We searched 11 WHO primary registries for Chinese trials records. The progress of each trial was analyzed. We searched for the full texts of result publications cited in the registration records. For completed trials without citations, we searched PubMed, Embase, Chinese Biomedical Literature Database (Chinese), China Knowledge Resource Integrated Database, and Chinese Science and Technology Periodicals Database for result publications. The search was conducted on July 14, 2009. We also called the investigators of completed trials to ask about results publication.

**Results:**

We identified 1294 Chinese trials records (428 in ChiCTR,743 in clinicaltrials.gov,55 in ISRCTN, 21 in ACTRN). A total of 443 trials had been completed. The publication rate of the Chinese trials in WHO primary registries is 35.2%(156/443).The publication rate of Chinese trials in clinicaltrials.gov, ChiCTR, ISRCTN, and ACRTN was 36.5% (53/145), 36.3% (89/245), 26.0%(9/44), and 55.6%(5/9), respectively. The publication rate of trials sponsored by industry(23.8%) was lower than that of sponsored by central and local government(31.7%), hospital(35.1%), and universities (40.7%). The publication rate for randomized trials was higher than that of cohort study and case-control study (33.2% versus 16.7%, 22.2%). The publication rate for interventional studies and observational studies was similar(33.4% versus 33.3%).

**Conclusion:**

The publication rate of the registered Chinese trials was low, with no significant difference between ChiCTR and clinicaltrials.gov. An effective mechanism is needed to promote publication of results for registered trials in China.

## Introduction

Trial results published in peer-reviewed journals are a major source of scientific information for health policymakers, patients, doctors, and researchers. However, non-publication of results and selective outcomes reporting make the available body of evidence incomplete and possibly biased. This can lead to duplicate research and funding, and to harm for patients whose health care was not informed by the best evidence [1]–[5]. Positive results are usually considered more interesting (and publishable) than negative results, and the results of many trials are under disclosure. Therefore, published trials together provide an incomplete picture of overall research results [6]–[22]. Clinical trial registration was thus established to improve the transparency of clinical trials, reducing selective outcome reporting.

In May 2007, the International Clinical Trials Registry Platform of the World Health Organization (WHO ICTRP) was established to offer an international standard for trial registration. ICTRP made trial registration the rule, not the exception. As of July 2009,10 WHO primary registries in Australia and New Zealand, the United Kingdom, China, India, Germany, Japan, the Netherlands, Iran, Sri Lanka, and Africa are approved by WHO ICTRP. Another registry, Clinicaltrials.gov, is approved by the International Committee of Medical Journal Editors (ICMJE)[10]. ICMJE accepts registration of trials in any of the WHO ICTRP primary registries, and WHO ICTRP accepts registrations approved by ICMJE [11]–[14]. FDAAA 801(section 801 of FDAAA, a United States public law covering clinical trial) also calls on the US National Institutes of Health to require that Clinicaltrials.gov entries be augmented with trial results within 12 months of trial completion or within 30 days of FDA approval (or clearance) of a new drug, biologic, or device (with the exception of phase I clinical trials and complementary and alternative medicine trials)[13], [15]. Our previous study showed that Chinese trials are registered not only in the Chinese Clinical Trial Register (ChiCTR), but also in other WHO ICTPR primary registries or ICMJE-approved registries[16].

Many trials are conducted in China. The State Food and Drug Administration (SFDA) of China receives about 1,250 applications for authorization of new trials and new drug applications each month [17]. Up to now, no published studies have explored the result publication rate in China after registration. We aimed to assess the result publication rate in the 10 WHO registries and to identify factors associated with publication.

## Materials and Methods

### Inclusion and exclusion criteria

We included trials we considered to be “sponsored by China”—that is, either the principal investigator, the main funder, or both were located in China. We excluded trials in which study participants were Chinese, but the trial was not sponsored by China.In this study, a Chinese trial could be either interventional or observational.

### Data collection

We searched 11 registries for trials sponsored by China: the Australian and New Zealand Clinical Trials Registry (ACTR), the International Standard Randomised Controlled Trial Number Register (ISRCTN), the Chinese Clinical Trial Registry (ChiCTR), the Iranian Registry of Clinical Trials (IRCT), the German Clinical Trials Register (DRKS), the Japan Primary Registries Network, the Netherlands National Trial Register (NTR), the Sri Lanka Clinical Trials Registry (SLCTR), the Clinical Trials Registry – India (CTRI), the Pan African Clinical Trial Registry (PACTR), and Clinicaltrials.gov.

All records in ChiCTR were included in our analysis, and we used “China” as a key word in searching the other 10 registries for potentially eligible trials. The search was conducted on July 14, 2009.

### Data extraction

We designed a program using the programming language Python to extract information about clinical trials. The program first automatically fetches web pages of clinical trials from registration centers and stores them in separate HTML files. Then it utilizes specific tags and key words in HTML files to locate each of the registration items, and extracts them. Finally, this program compiles the extracted data and exports it into an Excel table for further analysis. The first search was conducted on December 31, 2009 and included 920 trial records. To guarantee the reliability and reproducibility of the program, we checked the 920 records manually. Our check confirmed that the program could extract the data correctly. The rate of agreement is 100%. The final search was conducted on July 14, 2009.

**Figure 1:**
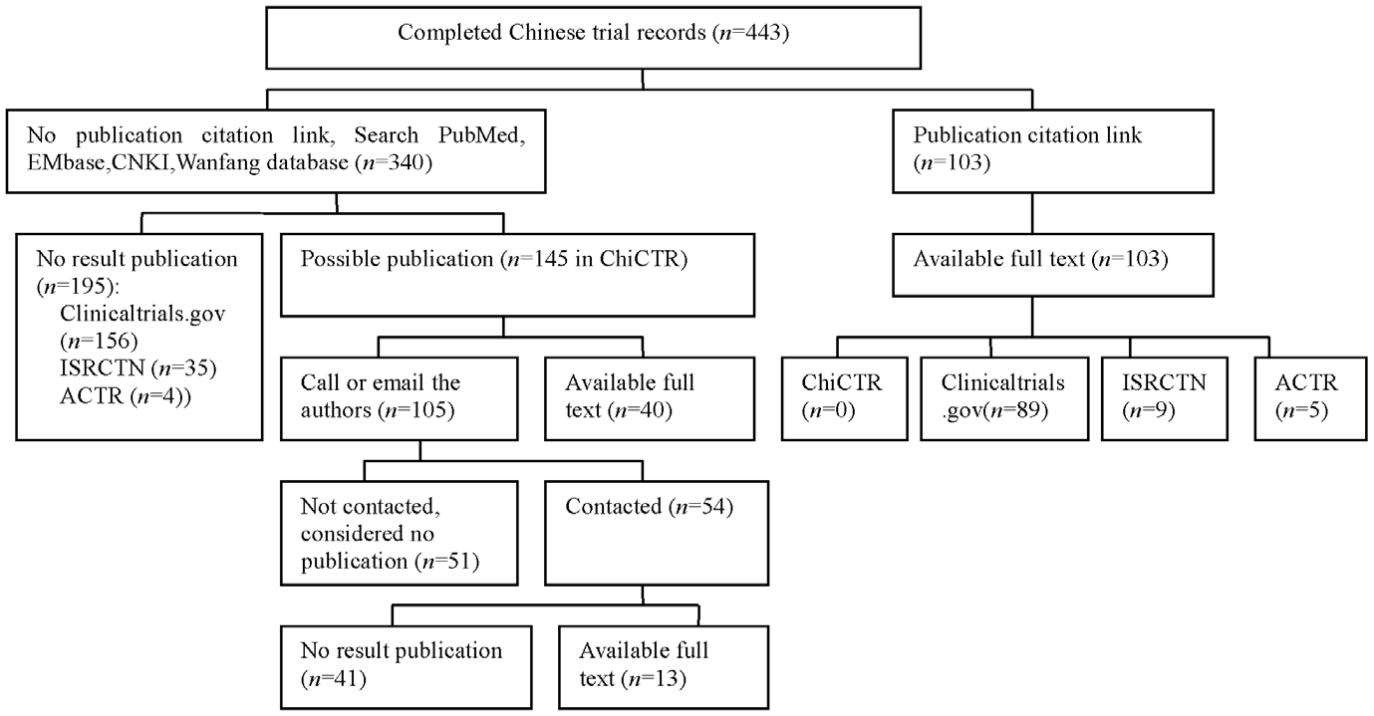
Means of obtaining full text of completed trials.

The progress status of a trial was indexed as completed, ongoing, terminated, or suspended. To find the full text of each completed trial, we proceeded as follows (see figure 1)

1. We looked for links to published results in the registry.
2. If there were no links to published results in the registry, we used key words from the record to search systematically for the published study in PubMed (English language, 1966 to July 14, 2009), Embase (English language, 1974 to July 14, 2009), CNKI (Chinese language, 1979 to July 14, 2009), Wanfang database (Chinese language, 1990 to July 14, 2009), and CBM (Chinese language, 1978 to July 14, 2009). We identified a citation as the result publication by matching registration number, the authors, institute, interventions, participants, outcome measures, study design, and study start date in the registration record.
3. If contact information was available in the registry, we called or emailed the investigators to ask for results publication information.

Two authors (Liu XM and Yin SL) devised the search strategy. Two authors (Yin SL and Song SQ) independently searched the databases, and shared the work of calling or emailing the authors. Differences were resolved by discussion among all the authors.

### Publication of trial result

The publication rate (the proportion of included trials with result publications) was calculated. To examine the associations between trial characteristics and publication rate, we calculated the publication rate for trials at different stages, and with various sponsor types, study designs, and intervention types. The trial stage was categorized as completed, ongoing, terminated, or suspended; sponsor types were industry, central or local government body, hospital, or university; study designs were observational, interventional, randomized, and non-randomized; interventions were biological, behavioral, dietary supplement, device, procedure, drug, gene transfer, other, and not specified.

After a trial is completed, time is needed to analyze the data and write the paper. So we conducted a sensitivity analysis in publication rate by excluding trials completed after July 2008 from our analysis.

We also calculated the registration number reporting rate for the publication full texts.

### Statistical analysis

Publication rates were described as proportions. Data were analyzed by EXCEL software.

## Results

### Search results

#### Number of registered trials

We initially screened 1294 records of Chinese trials in clinicaltrials.gov. After we carefully read the registration information, we excluded 551 records with a recruitment location in China, but no investigators affiliated with China administratively.

We finally included 1247 records (743 in clinicaltrials.gov, 428 in ChiCTR, 55 in ISRCTN.COM, and 21 in ACTR). No records Chinese trials were found in the Indian, German, Japanese, Dutch, Sri Lankan, Iranian, or Pan African registries.

#### Result publication

As of July 14, 2009, 35.5% (443/1247) of the registered Chinese trials had been completed. The publication rate of the completed trials was 35.2% (156/443). The publication rate of the completed trials in ACTR was the highest of the 4 registries, and similar publication rates in ChiCTR and clinicaltrials.gov were found. As of July 14, 2008, 332 trials were completed. The publication rate was a little higher at 39.8% than that of the trials completed before July 14, 2009 (see Table 1). Among the 145 completed trials in ChiCTR, we found the published results for 40 through our systematic search. We called the investigators of the other 105 trials. A total of 51 trial investigators were contacted; the other 54 investigators could not be contacted because the telephone number in the registry was wrong, the mobile phone was off, or there was no answer. The investigator of one trial stated that the results were published but provided no details. We did not find the publication.

**Table 1:** Publication rate of the completed Chinese trials in WHO/ICMJE primary registries.

Registries	Trials completed before July 14, 2009(*n* = 443)			Trials completed before July 14, 2008(*n* = 332)		
	Publication		Publication rate (%)	Publication		Publication rate (%)
	Yes	No		Yes	No	
ChiCTR	53	92	36.5	40	61	39.6
Clinicaltrials.gov	89	156	36.3	78	118	39.8
ISRCTN	9	35	26.0	9	17	34.6
ACTR	5	4	55.6	5	4	55.6
Total	156	287	35.2	132	200	39.8

The call investigation found result publications for 13 trials. The main reasons investigators gave for non-publication were that the manuscript was in preparation (21 trials), that there was no intention to publish results (7 trials), or that the manuscript had been rejected by journals (2 trials). The investigators of 7 trials said that although the trials were indexed as “completed,” these trial were not in fact completed.

We could not get the email or telephone number of the investigators for the completed Chinese trials in clinicaltrials.gov because contact information is only displayed in that registry when the study is recruiting subjects. We obtained all the full texts of the included trials in ACTR and ISRCTN. Therefore, we did not contact the investigators for trials in these three registries.

### Factors association with the result publication

#### Sponsorship

The publication rate for the Chinese trials sponsored by industry was the lowest of the four sponsor types at 23.8%, while the publication rate for Chinese trials sponsored by universities was the highest, at 40.7% (table 2).

**Table 2:** The publication rate and sponsorship of the completed Chinese trials in ChiCTR, clinicaltrials.gov, ISRCTN, ACTRN.

Sponsorship	ChiCTR (*n* = 145)			Clinicaltrials.gov (*n* = 245)			ISRCTN(*n* = 44)
	Publication		Publication rate (%)	Publication		Publication rate (%)	Publication
	Yes	No		Yes	No		Yes
Industry	1	1	50.0	14	44	24.1	0
Central and local government	2	9	18.2	14	23	37.8	9
Hospital	45	77	26.2	16	24	40.0	0
University	5	5	50.0	45	65	40.0	0

		ACTRN(*n* = 9)			Total (*n* = 443)		
	Publication rate (%)	Publication		Publication rate (%)	Publication		Publication rate (%)
No		Yes	No		Yes	No	
4	0	1	0	100	16	51	23.8
22	29.0	4	2	66.7	25	54	31.7
7	0	0	1	0	61	113	35.1
2	0	0	1	0	50	73	40.7

The publication rate for trials sponsored by industry in ChiCTR was higher than that of trials sponsored by governments or hospitals (table 2). However, the available sample was small (only 2 trials were sponsored by industry, with 1 published and 1 unpublished result). The publication rates for government-sponsored trials in ChiCTR, clinicaltrials.gov, and ISRCTN were all low (table 2).

#### Publication rate by trial stage

Of the 743 Chinese trials in clinicaltrials.gov, 245 (33.0%) had been completed, 23 (3.1%) had been terminated, 5 (0.7%) had been suspended, and 470 (63.2%) were ongoing. The publication rate of the competed, ongoing, suspended, and terminated trials was 36.3% (89/245), 3.6% (17/470), 10% (1/5), and 8.7% (2/23), respectively.

Of the 145 completed trials in ChiCTR, 124 (85.5%) were retrospectively registered (registered between trial start and result publication), and 21 (29.8%) were prospectively registered. The publication rate of retrospectively registered trials (29.8%) was higher than that of prospectively registered trials (14.3%).

#### Publication rate by study design

The publication rate of randomized controlled trials was higher than those of cohort and case-control studies. The result publication rates for trials of preventative and health services research were higher than those of supportive care, treatment and diagnostic trials. The publication rate of interventional and observational studies was similar (Table 3).In Clinicaltrials.gov, publication rate of randomized controlled trials was higher than non-randomized controlled trials, publication rate of interventional studies was higher than observational studies; while in ChiCTR and ACTRN, it was contrary.

**Table 3:** Publication rate of study type and design in clinicaltrials.gov (*n*, %).

Study design	Clinicaltrials.gov		Publication rate (%)	ChiCTR		Publication rate (%)	ISRCTN
	Publication			Publication			Publication
	Yes	No		Yes	No		Yes
Randomized controlled trial	67	108	62.0	27	70	27.8	8
Non-randomized controlled trial	10	15	40.0	5	12	29.4	1
Cohort study	2	8	20.0	1	5	16.7	-
Case-control study	1	9	10.0	1	3	25.0	-
Study type							-
Prevention	13	12	52.0	3	7	30.0	-
Health services research	1	1	50.0	-	-	-	-
Supportive care	3	4	42.9	-	-	-	-
Treatment	60	103	36.8	21	66	24.1	-
Diagnostic	2	6	25.0	2	3	40.0	-
Interventional or observational							
Interventional	80	128	38.5	23	64	26.4	9
Observational	9	28	24.3	6	4	60.0	-

	Publication rate (%)	ACTRN			Total		Publication rate (%)
		Publication		Publication rate (%)	Publication		
No		Yes	No		Yes	No	
29		3	4	42.9	105	211	33.2
2	-	-	-	-	16	29	35.6
2	-		-	-	3	15	16.7
2	-	2	0	100.0	4	14	22.2
-	-	-	-	-			
-	-	-	-	-	16	15	51.6
-	-	-	-	-	1	1	50.0
-	-	-	-	-	3	4	42.9
-	-	-	-	-	81	169	32.4
-	-	-	-	-	4	9	30.8
							
33	-	3	4	42.9	115	229	33.4
2	-	2	0	100.0	17	34	33.3

#### Publication rate by intervention type

No classification of the interventions of trials was provided in ChiCTR, so we only analyzed the publication rates for different interventions in clinicaltrials.gov. The publication rate for biological research was the highest (Table 4). The number of the drug trial was the most, but its publication rate was the lowest (Table 4).

**Table 4:** The publication rate by interventions for Chinese trials in clinicaltrials.gov (*n*,%).

Interventions	Publication		Total	Publication rate (%)
	Yes	No		
Biological	6	1	7	85.7
Behavioral	4	3	7	57.1
Other	4	3	7	57.1
Dietary Supplement	2	2	4	50.0
Device	9	11	20	45.0
Procedure	16	20	36	44.4
Drug	43	96	139	30.9
Unclear	5	19	24	20.8
Gene Transfer	0	1	1	0.0
Total	89	156	245	-

### Registration number reporting in full text of result publications

We obtained the full text of result publications for 62.2% (97/156) of the completed trials. Only 25.8% (25/97) of the result publications reported registration number.

Of the 38 result publications in ChiCTR, Only 1 (a doctoral dissertation) reported a registration number.

Of the 45 result publications in clinicaltrials.gov, 21publications for 20 trials reported registration number. Apart from one result publication in the Chinese-language *National Medical Journal of China*, the publications that reported registration number were all published in English-language journals outside China.

Nine publication full texts for the 9 trials in ISRCTN were obtained. Only two publications for 2 trials reported registration numbers. Five publications for 5 trials reported informed consent and the name of the ethical review body.

We obtained 5 publication full texts for 5 trials in ACTR. Two of the publications reported registration numbers.

## Discussion

The publication rate of completed, registered Chinese trials was only35.2%.Even for trials had been completed since at least one year, the publication rate was also only 39.8%. The publication rate of registered Chinese trials is lower than the average (46%) for all trials registered in clinicaltrials.gov. The publication rate is similar in clinicaltrials.gov and ChiCTR. It was also similar to the low publication rates found in previous studies [23]. A meta-analysis showed that the publication rates for trials in inception cohort, regulatory cohort, abstract cohort, and manuscript cohort varied from 60% to 93% for significant results and from 20% to 86% for non significant results [23].

In 2005 ICMJE recommended that investigators be required to prospectively register randomized controlled trials, but only a few Chinese journals have implemented this policy. Accordingly, we found that only 25.8% of result publications reported a registration number. In contrast, Mathieu et al. found recently that among articles indexed by MEDLINE in cardiology, rheumatology, and gastroenterology, 76.4% reported the registration number [24]. This may be because higher impact journals are more likely to require registration number reporting. Therefore, we suggest that all journals should require submitted papers to include a registration number.

### Factors association with non publication

The low publication rate we found indicates that the current trial registration system does not promote publication effectively. It does, however, provide a convenient means to identify non publication. We found several factors associated with non publication:

#### Sponsorship

Trials sponsored by industry were less likely to have result publications than trials with other funding sources, indicating that conflict of interest may be a factor in non publication. This accords with the findings of Ross et al., who report that the publication rate of trials sponsored by industry is lower than those with non-industry support (40% versus 56%) in clinicaltrials.gov. However, Ross et al. found that the publication rate for trials primarily sponsored by the National Institutes of Health (NIH) is also just 40% (30/74) [15]. In contrast, Dickersin et al. report that the publication rate for 198 clinical trials funded by the NIH is 93%[21]. Ramsey et al. find that just 5.9% of the oncology trials sponsored by industry in clinicaltrials.gov published results indexed by PubMed[25]. Liebeskind et al. report that the publication rate of trials on efficacy of acute stroke treatments conducted between 1955 and 1999 is at 76% (136/178)[26]. A systematic review of 6 studies of funding sources and publication status shows that trials funded by industry are less likely published than other funded sources [27].

#### Study design

We find that the publication rate for randomized controlled trials is higher than for case control and cohort studies. Easterbrook et al. also find that randomized controlled trials have a lower risk of non publication than observational studies [20].

#### Intervention type

Though drug trials are the most numerous among those registered, their publication rate is lower than those of biological, behavioral, other, dietary supplement, and device procedures. This suggests that the publication bias for drug trials is more serious than for other interventional trials in China. A similar effect is also found in clinical trials involving acute stroke[26].

#### Termination/suspension

Only about one-tenth of terminated or suspended trials had result publications, suggesting a high risk of non publication for these trials.

In our telephone interviews, the reasons cited by investigators for non publication included that the manuscript had been rejected by the journal, and a lack of time for writing. This is consistent with previous reports. Kryzyzanowska et al. interviewed 34 authors who had not published results, and found that common reasons for failure to publish were lack of time, funds, or other resources [22]. Other studies show that the main reason for failure to publish results is that investigators lack the time to submit the full manuscript to a peer-reviewed journal [21], [28]–[30].

### Reducing non publication

The Food and Drug Administration Amendment Act of 2007 requires investigators to submit results to clinicaltrials.gov. Investigators who do not comply can be fined as much as $10,000 per day or have their federal research funds withheld or recovered[31]. The law may work. The publication rate of trials sponsored by NIH (46%) is higher in clinicaltrials.gov[15] than that of Chinese trials (35%) registered in WHO primary registries in our study.

A recent study concludes that three proposed remedies, financial disclosure, reporting standards, and trial registries, failed to control sponsorship bias[32]. The low publication rate of Chinese trials registered in clinicaltrials.gov or ChiCTR suggests that trial registration alone, without a requirement of result publication, does not improve publication rate. Non publication was still common in trials registered in clinicaltrials.gov [15], [24].

Although trial registration is becoming more common in China, no systematic enforcement of the result posting requirement has yet been established for WHO primary registries. Posting results in the registry and publishing in peer reviewed journals should be encouraged to avoid a low result publication rate.

### Limitations

Our study has some limitations. First, it only investigates the publication rate of registered trials, so the time lag between trial and publication, as well as any selective outcome reporting bias, remain uncertain. Second, we are unable to follow up with all of the lead investigators on completed, unpublished trials, since no email addresses or telephone numbers are posted for completed Chinese trials in clinicaltrials.gov, and investigators of 54 trials in ChiCTR could not be contacted. So the publication status and reasons for non publication of these trials is unknown. Third, although we use a comprehensive search strategy to find the result publications, some publications are likely omitted. In order to more fully understand publication status in registered Chinese trials, further research will be need to examine the time lag between trial and publication, and the comparative publication rates for positive and negative results.

### Conclusion

Many previous studies have examined potential sources of non publication, abbreviated publication, selective outcomes reporting, and time lag bias[26], [29]–[31], [32]. Just two studies[15], [31] have investigated publication rate through registration[15], [32]. This is the first study to report on the publication rate of registered Chinese trials.

Non publication trial is a more serious problem in China than in other countries. Mandatory result posting in a registry is needed to promote result publication. Result publication policies should especially focus on result publication of industry-sponsored trials, poorly designed trials, and terminated or suspended trials. Regular, complete, and transparent reporting is urgently needed to ensure the integrity of Chinese science.
